# Superoxide Radical Dismutation as New Therapeutic Strategy in Parkinson’s Disease

**DOI:** 10.14336/AD.2017.1018

**Published:** 2018-08-01

**Authors:** Federica De Lazzari, Luigi Bubacco, Alexander J Whitworth, Marco Bisaglia

**Affiliations:** ^1^Molecular Physiology and Biophysics Unit, Department of Biology, University of Padova, 35131 Padova, Italy.; ^2^Medical Research Council Mitochondrial Biology Unit, University of Cambridge, Cambridge Biomedical Campus, Cambridge, CB2 0XY, UK.

**Keywords:** antioxidants, oxidative damage, Parkinson’s disease, SOD-mimetics, superoxide dismutases

## Abstract

Aging is the biggest risk factor for developing many neurodegenerative disorders, including idiopathic Parkinson’s disease (PD). PD is still an incurable disorder and the available medications are mainly directed to the treatment of symptoms in order to improve the quality of life. Oxidative injury has been identified as one of the principal factors involved in the progression of PD and several indications are now reported in the literature highlighting the prominent role of the superoxide radical in inducing neuronal toxicity. It follows that superoxide anions represent potential cellular targets for new drugs offering a novel therapeutic approach to cope with the progression of the disease. In this review we first present a comprehensive overview of the most common cellular reactive oxygen and nitrogen species, describing their cellular sources, their potential physiological roles in cell signalling pathways and the mechanisms through which they could contribute to the oxidative damage. We then analyse the potential therapeutic use of SOD-mimetic molecules, which can selectively remove superoxide radicals in a catalytic way, focusing on the classes of molecules that have therapeutically exploitable properties.

Parkinson’s Disease (PD) represents a common neurodegenerative disorder that affects approximately 1-2% of population over the age of 65 years. The parkinsonian symptoms include bradykinesia, resting tremors, muscular rigidity, postural instability, and gait impairment [1]. The neuropathology of PD is characterized by a preferential neuronal loss of dopaminergic cells within *Substantia Nigra pars compacta* (SNpc) and by the presence of inclusions called Lewy bodies in the surviving neurons [2]. Although most PD cases are sporadic, the identification of familial forms of the syndrome, accounting for 5-10% of PD cases, has helped to shed some light on the cellular pathways involved in the pathology. Genetic manifestations of the disease are classified into dominant or recessive forms, according to the pattern of inheritance. α-Synuclein and LRRK2 contribute to the former, while Parkin, PINK1, and DJ-1 are associated to the latter. α-Synuclein is a major constituent of Lewy Bodies aggregates, while LRRK2 is a protein kinase involved in multiple cellular processes, including neurite outgrowth and synaptic morphogenesis. In contrast, Parkin, PINK1, and DJ-1 are all primarily implicated in mitochondrial health. Parkin, an E3 ubiquitin ligase, and PINK1, a serine-threonine protein kinase, cooperate to remove damaged mitochondria, whilst DJ-1 is redox-sensitive protein which protects mitochondria and cells against oxidative stress via an unknown mechanism [1]. Even though the aetiology of sporadic PD remains elusive, there is evidence that oxidative injury plays a significant role in the development of the disease [3,4]. Notwithstanding the intense research performed in the field, at present there is no cure for PD, but only symptomatic treatment, principally based on the administration of L-DOPA, the dopamine precursor [5].

## Oxidative Injury and PD

**Figure 1.**
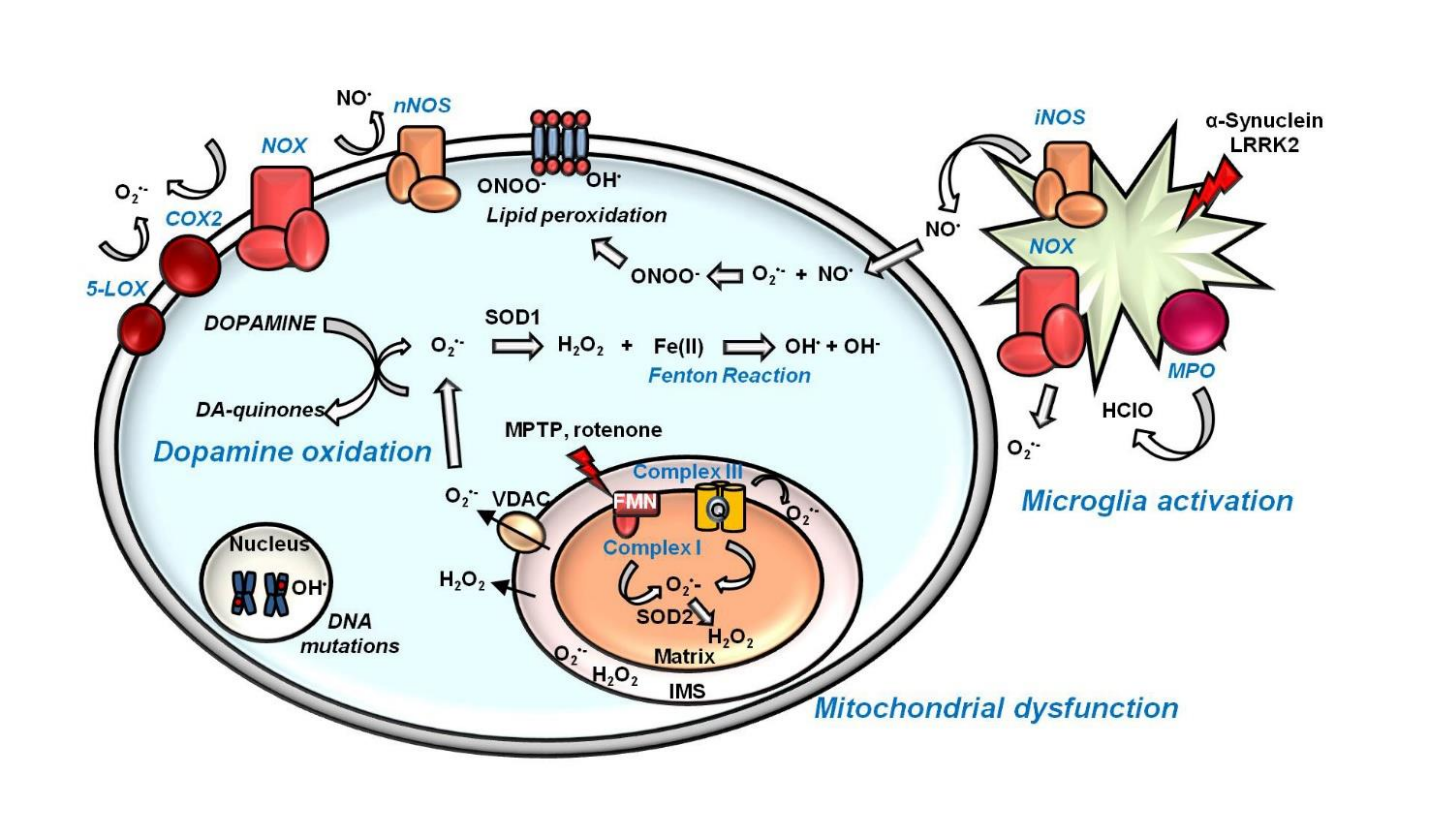
Oxidative injury and PD Mitochondrial dysfunction, dopamine metabolism and neuroinflammation co-participate in promoting oxidative damage in dopaminergic neurons. Complexes I and III of the mitochondrial electron transport chain are the main sources of O_2_^•-^ inside cells. The PD-related toxins MPTP and rotenone are complex I inhibitors. Chronic activation of microglia represents a further mechanism of O_2_^•-^ and NO^•^ radical’s generation through the action of NADPH-oxidase (NOX) and inducible nitric oxide synthase (iNOS), respectively. In activated microglia, myeloperoxidase (MPO) is responsible for the formation of HClO. The PD-associated proteins α-Synuclein and LRRK2 contribute to microglia activation. Specific to dopaminergic neurons, the cytosolic oxidation of dopamine to dopamine-quinones leads to the production of the O_2_^•-^. Cytosolic O_2_^•-^ is transformed by the action of superoxide dismutase 1 (SOD1) in H_2_O_2_, which can produce the highly toxic radical HO^•^ through the Fenton reaction. Otherwise, O_2_^•-^can react with NO^•^ to form the very reactive molecule ONOO^-^. (FMN: flavin mononucleotide; IMS: intermembrane space; nNOS: neuronal nitric oxide synthase; VDAC: voltage-dependent anion channels).

A special interest has been attributed to the role of oxidative damage in neurodegeneration, since the discovery that SN in PD patients presents high levels of oxidized DNA, proteins and lipids and lower levels of glutathione (GSH) [6], suggesting that the excessive production of reactive oxygen species (ROS) and reactive nitrogen species (RNS) might contribute to neuronal death. Oxidative injury derives from an imbalance in ROS/RNS homeostasis, due to an excessive production of these species or impairment in their detoxification. As represented in Fig. 1, multiple causes seem to trigger oxidative damage in PD. Indeed, mitochondrial dysfunction, dopamine metabolism and neuroinflammation are all thought to be involved in this stressful condition [3].

### Mitochondrial dysfunction

Because of its high-energy demand, brain functions strongly depend on mitochondrial activity. Even when the mitochondrial electron transport chain (ETC) works efficiently, the redox reactions that occur during oxidative phosphorylation render each electron carrier in the ETC prone to react with molecular oxygen, producing ROS [7]. Superoxide anion (O_2_^•-^) and hydrogen peroxide (H_2_O_2_) represent the major ROS species produced during oxidative phosphorylation, and their generation is substantially increased when ETC is impaired in dysfunctional mitochondria. The discovery that MPTP (1-methyl-4-phenyl-1,2,3,6-tetrahydropyridine), a contaminant of a synthetic opiate, can cause parkinsonism through its neurotoxic metabolite, 1-methyl-4-phenylpyridinium (MPP+), which acts as a complex I inhibitor, revealed a role of mitochondria in PD pathology [8]. Successively, other inhibitors such as rotenone, pyridaben, and fenpyroximate have been found, supporting the role of mitochondrial complex I dysfunction in oxidative stress [9]. Accordingly, *post-mortem* analysis on PD patients showed that mitochondria frequently present a lowered complex I activity [10-12]. Besides complex I, also complex II and III can participate in ROS generation, since their components can promote oxygen reduction, leading to O_2_^•-^ [7]. Further support to the involvement of mitochondria in PD and ROS production derives from the finding that the PD-associate proteins Parkin, PINK1 and DJ-1 are involved in mitochondrial functioning. Studies in fruit flies clearly demonstrated that PINK1 and Parkin participate in controlling mitochondrial morphology and homeostasis [13-15]. Moreover, it has been reported that overexpressing DJ-1 pathological mutants in neuronal cells results in mitochondria fragmentation [16], and that, under oxidative conditions, DJ-1 translocates into mitochondria where it exerts its protective function, promoting cell survival [17]. Interestingly, DJ-1 has been reported to directly bind subunits of the mitochondrial complex I, regulating its activity [18]. In conclusion, mitochondria are critical organelles for the maintenance of brain functionality and their dysfunction seems to be deeply connected to increased oxidative conditions, which, in turn, might render dopaminergic neurons highly susceptible to cell death.

### Neuroinflammation

The brain has a strictly governed immune response, which relies on the presence of resident cells: microglia and astrocytes. Microglial cells, in particular, are the principal actors of the immune response within the central nervous system [19]. Under physiological conditions, microglia present a quiescent phenotype and act to maintain brain homeostasis through the release of neurotrophic and anti-inflammatory factors. Once activated, microglial cells initiate the immune response, stimulating the restoration of tissue homeostasis. Normally, this protective response is concluded after the triggering stress factor has been eliminated. However, if the stimulus is not eradicated, the chronic activation of microglia leads to the establishment of a deleterious permanent inflammatory state that leads to the accumulation of toxic ROS and RNS [19]. The principal ROS/RNS species released by activated microglia are O_2_^•-^ and NO^•^. The former is produced by nicotinamide adenine dinucleotide phosphate oxidase (NADPH-oxidase), while the latter is synthesized by the nitric oxide synthase (NOS), enzymes that participate to mount the immune response [20]. The importance of microglia dysfunction in PD pathology has been first reported in 1988, when McGeer and colleagues revealed the presence of reactive microglia in SNpc of *post-mortem* brain tissues from PD patients [21], and the role of neuroinflammation in PD is currently well accepted [22]. Neuromelanin, the dark pigment found in SN dopaminergic cells, has been proposed as an endogenous microglial activator related to PD. Although its role is still controversial, it has been shown that neuromelanin released from dying neurons operates as a microglial trigger, through NADPH-oxidase activation [23]. This phenomenon promotes a feed-forward mechanism that generates a chronic state of inflammation contributing to the further propagation of the neuropathology. As for mitochondria dysfunction, the involvement of neuroinflammation in PD is also supported by the finding that the PD-associated proteins α-Synuclein and LRRK2 contribute to or modulate microglia activation [24]. In fact, extracellular α-Synuclein aggregates, released by dying neurons, are internalized by microglia, leading to NADPH-oxidase activity and, therefore, to ROS and RNS production [25, 26], while LRRK2 seems to positively regulate the pro-inflammatory response, since its downregulation is associated to a reduced state of inflammation [27, 28]. In conclusion, even though it is still unclear whether neuroinflammation is causative of PD or a consequence of the disorder, its contribution to ROS/RNS production and to the progression of the disease is now well accepted.

### Dopamine metabolism

The underlying cause of the selective dopaminergic cell loss in PD is still an open question. Dopamine itself has been suggested to contribute to the preferential vulnerability of dopaminergic neurons through the generation of oxidative injury [29, 30]. While dopamine is a stable molecule inside synaptic vesicles, due to the low pH value, its instability at physiological pH, renders the molecule prone to oxidation at its electron-rich catechol moiety. This leads to the production of hydrogen peroxide and superoxide anion and the formation of highly reactive dopamine-derived quinones (DAQs), which participate in nucleophilic addition reactions [30]. At physiological pH, the cysteine residues of proteins are among the strongest cellular nucleophiles able to interact with DAQs. As these residues are often localized in the active sites of proteins, their interaction with DAQs could be responsible for the alteration of protein functionality, which can result in enzyme inactivation [30]. In addition to proteins, DAQs have been reported to be genotoxic, affecting DNA stability. Their preferential targets are guanine bases, since its N7 position is easily accessible. The formation of DNA adducts is the first step to DNA alterations and mutations, which can finally lead to apoptosis [31]. Through its amino moiety, dopamine has been shown to interact with lipid hydroperoxides produced during lipid peroxidation leading to the formation of adducts that could account for the selective cytotoxicity of dopaminergic neurons. Indeed, dopamine adducts derived from polyunsaturated fatty acids are highly reactive and can interfere with normal cellular processes, promoting cytotoxicity. For example, hexanoyl dopamine (HED), an arachidonic acid-derived adduct, has been shown to cause severe cytotoxicity in the human dopaminergic neuroblastoma SH-SY5Y cell line [32].

While PD pathology has been reported to affect also non-dopaminergic cells, particularly during the late stages, and, even though not all dopaminergic neurons are equally affected during PD progression [33], the high toxic potential owned by dopamine itself could contribute to make dopaminergic neurons more sensitive to oxidative damage.

## Reactive oxygen species

The principal cellular ROS are the superoxide radical O_2_^•-^, hydrogen peroxide H_2_O_2_ and hydroxyl radical OH^•^. Among them both O_2_^•-^ and OH^•^ are free radical species, since their orbitals possess one unpaired electron. The superoxide anion O_2_^•-^ is usually referred as the “primary” ROS, because it is capable of interacting with other molecules to form “secondary” ROS [34]. However, O_2_^•-^ is not highly reactive toward cellular macromolecules such as proteins, nucleic acids and sugars [34]. The superoxide radical has been described to react with proteins, changing their redox state without damaging their structure [35]. Its toxic potential relies principally on the fact the most “secondary” ROS derive from it. Normally, O_2_^•-^ is readily detoxified by superoxide dismutases (SODs), which, in turn, release H_2_O_2_ and O_2_. H_2_O_2_ is inert by itself, but can react with O_2_^•-^ and iron via Haber-Weiss reaction generating the harmful HO^•^.

$${\text{Fe}(\text{II})+H}_{2}O_{2}{\to \text{Fe}(\text{III})+\text{HO}}^{•}{+\text{OH}}^{-}(\text{Haber-Weiss Reaction})$$

The hydroxyl radical has, *in vivo*, a very short half-life of 10^-9^ s, so that it acts near the site of its formation [20]. This radical is currently considered the most reactive one and the prevention of its synthesis represents the principal protective system [36]. Indeed, HO^•^ possesses a high damaging potential, considering that it avidly reacts with DNA, RNA, proteins and lipids, promoting cytotoxicity. For example, the interaction of HO^•^ with membrane lipids, induces a reaction called hydrogen abstraction, which generates carbon radicals, which can react with molecular oxygen to produce a lipid peroxyl radicals. These species can then interact with other lipids in close proximity, propagating the reaction and inducing lipid peroxidation [37]. These modifications impact on membrane fluidity and permeability, properties required for optimal cell integrity. Lipid peroxidation is further induced by redox active metal ions such as iron, which forms alkoxyl and peroxyl radicals. Specifically, reduced iron promotes alkoxyl radicals, while the oxidized ion induces peroxyl radicals. Both molecules participate then in lipid peroxidation, exacerbating the state of oxidative injury [37].

Another reactive “secondary” ROS, generated under inflammation by the enzymatically-controlled interaction of H_2_O_2_ with the chloride anion, is hypochlorite (HOCl), a ROS molecule that has also been linked to lipid peroxidation [38].

### Mitochondrial ROS production

Inside cells, mitochondria represent the principal source of O_2_^•-^, with complex I and III being the major sites of its production. The O_2_^•-^ formed at the level of Complex I is released in the matrix, where it is retained because of it charged state [20]. Here it is converted into H_2_O_2_ by the action of SOD2. Complex I is the starting point for electron flux in the respiratory chain. From NADH, electrons are accepted by flavin mononucleotide (FMN) and flow to seven iron-sulphur centres, finally arriving to co-enzyme Q (CoQ). O_2_^•-^ is formed by interaction of molecular oxygen with reduced FMN and this reaction is favoured by high NADH/NAD^+^ ratios in the matrix. Complex I can induce ROS production through an alternative mechanism which depends on reverse electron transport, a phenomenon that induces a reversion of the electron flux and that has been recently linked to oxidative damage in ischemia-reperfusion injury [39]. When complex II utilizes succinate as substrate, under low adenosine diphosphate (ADP) concentrations, the reduced form of quinone can reduce FMN in complex I, generating O_2_^•-^. Although the precise site of production is not clear, this process can be inhibited by rotenone, suggesting that O_2_^•-^ synthesis occurs at the FMN site [40].

Complex III, whose physiological activity consists of the transfer of electrons from ubiquinol to cytochrome c, is considered the second site of ROS production. The functional units of this complex are composed of two cytochrome b subunits forming a pocket into which the ubiquinol can diffuse, transferring electrons to cytochrome c. Through this so-called “Q-cycle”, complex III contributes to the hydrogen proton gradient required for ATP synthesis. Disruption of Q-cycle, for example through the inhibitor antimycin, drives O_2_^•-^ formation *via* interaction of oxygen with ubisemiquinone at the Q_o_ site of complex III. Differently from what described for complex I, O_2_^•-^ released by complex III is reversed both within the matrix and in the intermembrane space. SOD1 in the intermembrane space detoxifies O_2_^•-^ generating H_2_O_2_, which can passively diffuse through the outer mitochondrial membrane reaching the cytosol. However, O_2_^•-^ has also been reported to cross the outer membrane through the voltage-dependent anion channels (VDAC) thus accumulating in the cytosol [41].

It is worth mentioning that mitochondrial ROS production has been mainly estimated using isolated mitochondria under non physiological conditions [40]. For example, Hansford and co-workers determined that mitochondrial H_2_O_2_ production depends on a high reduction of complex I and a concomitant elevated membrane potential, conditions achievable only under non physiological conditions [42]. Therefore, although frequently defined as the primary sites of ROS production, mitochondrial complex I and III seem to promote significant ROS accumulation only under pathological circumstances, when there are alterations in their activity due to genetic mutations in the complex-forming proteins or upon exposure to inhibitors, like rotenone or MPTP [40, 43]. Currently less than 0.1% of oxygen consumed by these organelles is estimated to be converted into H_2_O_2_ under physiological conditions [44]. In the frame of PD, mitochondrial dysfunction has been suggested to enhance ROS synthesis, inducing their accumulation, which, in turn, leads to cytotoxicity. As recently reviewed, the involvement of mitochondria in PD pathogenesis is supported by *post-mortem* analyses on PD patients and by genetic and toxin-based animal models of PD [30].

### Extra-mitochondrial ROS sources

Besides the role of mitochondria in ROS generation, other sources cooperate to the production of reactive species synthesis. For example, membrane-bound NADH-oxidases (NOX) account for conspicuous ROS production. In the central nervous system, NADH-oxidases are mainly expressed in microglia, but they are also found in neurons, astrocytes and several neurovascular regions [45]. These multi-subunit enzymes are assembled after stimulation, as a host-defence mechanism. Although the regulation of their catalytic activities remains unclear, their contribution to superoxide production is well established [46]. Several PD related stimuli, such as α-Synuclein oligomers, have been reported to trigger NOX stimulation promoting ROS development [47]. The role of NOX in PD is further supported by the observation that knockout mice for the glycosylate protein gp91^phox^, the heme binding subunit of NOX, present milder dopaminergic degeneration compared to wild-type littermates [48].

Another source of ROS is represented by the protein myeloperoxidase (MPO), one of the most abundant enzymes of the body responsible for the formation of HClO, from H_2_O_2_ and Cl^-^, to kill pathogens during the respiratory burst. Being a mannosylated protein, MPO can bind to microglia membranes via mannose receptor, potentiating the anti-microbial function of these cells [49]. Although mainly present in the azurophilic granules of neutrophils, MPO has also been detected in activated microglia of PD patients’ brain, suggesting a role for MPO in neuroinflammation [50]. Accordingly, a recent study has demonstrated, *in vivo*, the efficacy of a selective inhibitor of MPO, called AZD3241, when administered to PD patients for 8 weeks. Indeed, through positron emission tomography study, researchers found a reduction in activated microglia, supporting a role of MPO in neuroinflammation associated to PD [51].

The metalloflavoprotein xanthine oxidase represents another route of O_2_^•-^ synthesis, through nucleic acids degradation. The enzyme catalyses the oxidation of hypoxanthine to xanthine and xanthine to uric acid. This two-step reaction requires molecular oxygen, which is eventually reduced to O_2_^•-^ and H_2_O_2_. However, the involvement of xanthine oxidase in neurodegeneration is less understood [52].

Cyclooxygenase and lipoxygenases are key enzymes involved in the conversion of arachidonic acid to a variety of eicosanoids, such as prostaglandins, thromboxane, and leukotrienes, hormone-like molecules that regulate a wide range of physiological process like blood pressure, platelet aggregation, and inflammation [53]. Both cyclooxygenases and lipoxygenases promote the formation of radical species that can react with molecular oxygen, giving rise to peroxyl radicals [54]. Their involvement in the context of PD is supported by the observation that the inhibition of either 5-lipoxygenase or cyclooxygenase isoform 2 confers protection against MPTP-dependent dopaminergic degeneration in mouse SN [55, 56]. Interestingly, dopaminergic neurons constitute the predominant cellular type where cyclooxygenase 2 is expressed [57].

## Reactive nitrogen species

This class of molecules encompasses nitric oxide radical (NO^•^), peroxynitrite (ONOO^-^), nitrogen dioxide radical (NO_2_^•^) [52]. Nitric oxide (NO^•^) is a nitrogen reactive radical characterized by one unpaired electron. It has a half-life of few seconds in aqueous solution, but its half-life increases up to 15 s under low oxygen concentrations. Due to its uncharged state, nitric oxide can easily diffuse in the cytoplasm as well as permeate biological membranes [20]. Its biosynthesis depends on the nitric oxide synthase enzyme (NOS), which catalyses the conversion of L-arginine to nitric oxide and L-citrulline. Flavin adenine dinucleotide (FAD), FMN, NADPH, tetrahydrobiopterin are all essential cofactors and their absence leads to NOS uncoupling, with formation of O_2_^•-^ rather than NO^•^. There are three NOS isoforms with distinct functions: neuronal NOS and epithelial NOS, which are activated by calcium flux, and inducible NOS, which is calcium independent. Although its synthesis is associated to pathogen defence, its chronic stimulation leads to biological damages. Indeed, during immune responses or under oxidative stress, NO^•^ can combine with O_2_^•-^, producing peroxynitrite ONOO^-^, a highly oxidizing agent, which induces protein nitration [20]. Related to PD, nitrated α-Synuclein has been detected in Lewy Bodies [58] and this modification has been reported to reduce α-Synuclein membrane affinity [59]. Peroxynitrite is also a potent source of oxyradicals, since it is readily decomposed into OH^•^ and NO_2_^•^, independently from the presence of transition metals, potentiating oxidative stress [60]. NO^•^ has a wide range of targets, including mitochondria, proteins, and lipids. At the mitochondrial level, NO^•^ induces complex III inhibition exhibiting an antimycin-like activity [61]. Moreover, NO^•^ binds iron-sulfur cluster of cytochrome ?? oxidoreductase, reversibly inhibiting the enzyme [62]. In PD, microglia represent the principal source of NO^•^, through the inducible form of NOS enzyme. As aforementioned, upon stimulation, microglial cells mount the “respiratory burst” to kill pathogens. However, chronic microglia activation, with the perpetuation of the inflammatory condition, has been proposed to further exacerbate the oxidative injury, making dopaminergic cells more vulnerable to cell death.

Considering ROS and RNS species and their relative reactivity, it has been hypothesized that while “primary” ROS/RNS have weak cytotoxic potential, “secondary” ROS/RNS present more deleterious effects. The best way that cells have to protect themselves against “secondary” ROS/RNS reactivity is through their avoidance, which can be achieved by a strict control of “primary” species. As most if not all the “secondary” ROS/RNS derive from the superoxide radical, a tight control of its levels appears crucial to preserve cellular functionality.

## Physiological functions of ROS and RNS: the other side of the coin

Besides the role of redox active species in cellular injury, growing evidence now supports their function as stress-responsive mediators on cell signalling pathways. Differently from other cellular mechanisms, redox signalling exploits reactive molecules as messengers, which transfer the information through protein modification, rather than by receptor binding. Indeed, at physiological levels, H_2_O_2_ oxidizes the thiolate anion of cysteine residues, changing protein redox-state. Because cysteines are principally localized in protein active sites, this modification plays an important role in enzymatic catalysis, modulating signalling pathways by protein activation or deactivation according to the H_2_O_2_ target. Another important determinant for ROS and RNS activities relies on their compartmentalization. For example, target proteins of H_2_O_2_ as redox signalling mediator should localize near its production site. Indeed, proteins that are physiologically modified by H_2_O_2_ produced from membrane-bound NADH-oxidase are localized near the membrane [63].

H_2_O_2_ has been shown to participate to several cellular signalling cascades, including NF-kB transcription factor, which is a principal mediator of inflammatory responses. Schmidt and co-workers reported that overexpression of catalase, the enzymatic down-regulator of H_2_O_2_, negatively impacts NF-kB activation, while, in contrast, overexpression of SOD1, which stimulates H_2_O_2_ production by O_2_^•-^ dismutation, increases NF-kB activation, supporting a direct role of H_2_O_2_ in the inflammation signalling pathway [64]. Also, O_2_^•-^ has been shown to modulate the immune responses. In particular, O_2_^•-^ generated by mitochondria seems to regulate inflammation through inflammasome activation and cytokines release. In fact, Zhou and colleagues demonstrated that mitochondrial dysfunction due to complex I and III inhibition, leads to “nucleotide-binding domain, leucine-rich family and pyrin domain containing 3” (NLRP3) activation, suggesting a role for O_2_^•-^ in inflammasome formation. [65] Coherently, Heid and co-workers showed that the use of Mito-TEMPO, a mitochondrial targeted SOD-mimetic, reduced IL-1β secretion after nigericin or ATP exposure. IL-1β secretion is an early event in inflammasome assembling [66]. Reactive species have also been described to modulate gene expression. The nuclear transcription factor-erythroid 2-related factor 2 (Nrf2) is the master transcription factor for anti-oxidant responses and it drives the expression of more than 100 genes involved in cellular protection. Under basal conditions, Nrf2 is inhibited by kelch-like ECH-associated protein (Keap1), which destabilizes Nrf2, promoting its degradation. Importantly, Keap1 possesses highly reactive cysteine residues, which can be oxidized upon exposure to oxidative stimuli, removing Keap1 inhibitory activity. Hence, under oxidative conditions, through the action of redox mediators, Nrf2is transferred to the nucleus where it stimulates the expression of cytoprotective genes [67].

**Table 1:** Physiological functions ascribed to ROS and RNS.

ROS/RNS species	Physiological functions	Reference
**O_2_^•-^**	Inflammasome activation and cytokines release	[65, 66]
**H_2_O_2_**	Modification of protein activity. Modulation of signalling pathways (NF-kB) and gene expression (Nrf2)	[64, 67]
**NO^•^**	Vasodilatation, platelet aggregation, neuronal firing, synaptic plasticity, and neurotransmitter release	[68, 69]

Not only ROS but also RNS have fundamental physiological functions. For example, NO^•^ participates to regulate vasodilatation, platelet aggregation, neuronal firing, synaptic plasticity, and neurotransmitter release [68]. Nitric oxide is considered an atypical neurotransmitter, since it is not stored in synaptic vesicles and it does not bind to receptors. Its synthesis is induced after stimulation of N-methyl-D-aspartate (NMDA) glutamate receptors and it is released immediately, diffusing through the pre-synaptic membrane. The principal molecular activity is the induction of guanylyl cyclase enzyme that synthesizes guanosine 3′5′-cyclic monophosphate (cGMP) a second messenger involved in multiple signalling pathways. Interestingly, it has been reported that NO^•^, *via* cGMP, also mediates long-term potentiation and long-term depression, two mechanisms involved in learning and memory processes [69]. Table 1 summarizes the main signalling functions ascribed to ROS and RNS.

These data support a role for ROS and RNS in signalling cascades, evidencing a protective function, exerted under physiological concentrations and in specific cellular compartments. Indeed, ROS and RNS are now considered dual-faceted molecules, which not only act as damaging agents, due to their chemical nature, but also positively contribute to cell homeostasis. What differentiates ROS and RNS as signalling molecules or damaging agents depends on their concentrations. [63]. What is not still fully understood is whether all ROS/RNS species are involved in cell signalling. Many studies support the physiological function of O_2_^•-^, H_2_O_2_, and NO^•^ (Table 1), while for other “secondary” species, only detrimental actions are reported. In conclusion, the cellular effects of a reactive species should be measured considering its relative concentration and cellular location and the mechanisms involved in its production and clearance.

## Antioxidants in PD therapy

PD is still an incurable disorder. While the available medications only treat the symptoms of PD, drugs that can arrest, reduce and/or delay the death of dopaminergic neurons and reverse the effects of the disease are not yet available. The current pharmacological therapy to replace the loss of dopamine is mainly based on the administration of L-DOPA, a dopamine precursor that crosses the blood-brain barrier (BBB). Unfortunately, L-DOPA therapy often loses its efficacy after few years of treatment, emphasising the urgent need for additional pharmacological approaches. As it is clear from what is discussed above, oxidative damage participates in neuron degeneration in PD. It follows that antioxidants should represent a valuable therapeutic approach to cope with the disease progression. Nevertheless, as recently reviewed [70-72], the effects attained in most of the controlled clinical trials are rather disappointing with very modest results. Several reasons could account for these disappointing clinical outcomes and their knowledge could provide the rationale to design novel strategies. First, a good therapeutic candidate must be able to survive ingestion, cross the intestinal barrier and reach the blood circulation without being degraded too rapidly in the plasma or eliminated through the kidneys. Moreover, it must be able to pass through cell membranes and, even more importantly, to cross the BBB. So, it is possible that the antioxidants used until now in clinical trials were not effective in reducing ROS/RNS levels because the required effective concentration was never reached on the target. Unfortunately, this issue cannot be easily addressed *in vivo* because of technical problems in measuring ROS concentrations and their variations [73]. Plasma levels of the supplemented antioxidants can be more easily monitored, even though this information can neither be easily related to the drug levels in the brain and, in particular, in dopaminergic neurons, nor to the effective elimination of ROS/RNS. Moreover, subsequently to their assumption, antioxidants could evenly distribute inside the body while the oxidative injury is mainly restricted to a specific region such as dopaminergic neurons in SNpc. As a consequence, the local concentration of antioxidants in the region of interest could be inadequate to deal with specific and local needs. Another point that must be considered is that, in addition to being harmful when they are present in excess, ROS/RNS play also important physiological functions, mainly as signalling mediators. As previously described, it is now evident that they affect several cellular functions such as proliferation, metabolism, differentiation, and survival, participate to the anti-oxidant and anti-inflammatory response and to iron metabolism [74]. It follows that their substantial removal can result in toxic rather than protective effects and a valuable therapeutic strategy should be aimed at keeping oxidant species at around the functional concentration rather than try to totally remove them.

In addition to the abovementioned explanations, focused on quantitative aspects, a central issue concerning most of the antioxidant-based clinical trials is that the real nature of the radical species actually involved in the progression of PD has never been carefully considered. As considered in the first part of this review, when discussing about ROS/RNS and their scavengers, it is crucial to know which kind of ROS/RNS is involved, because their chemical reactivity is different as well as their cellular targets. In particular, while “secondary” ROS/RNS have been demonstrated to be much more reactive than the “primary” ones, it is also clear that most if not all of them derive from the superoxide radical. In this frame, a key pitfall of the previously tested antioxidant therapies may be that they did not specifically target what could represent the primary cause of oxidative stress, i.e. excessive superoxide anion production, but rather the downstream effects (production of hydrogen peroxide, hydroxyl radical, peroxynitrite…). This is likely to be the case, for example, of α-tocopherol (vitamin E), ascorbic acid (vitamin C), creatine and apocynin [75]. In our opinion, a treatment strategy for oxidative stress is likely to be more effective if it targets the origin of ROS generation. This issue could be tackled by the administration of a mixture of antioxidant molecules directed towards different ROS species with the potential caveat that also side effects may act in a synergistic way. Alternatively, superoxide anions might be a potential therapeutic target for new drugs. In other words, antioxidant molecules, capable of decreasing superoxide radicals, could represent a potential therapeutic strategy to counteract the progression of PD.

## Superoxide radical dismutation and therapeutic implications

For their ability to convert superoxide radicals to molecular oxygen and hydrogen peroxide, Superoxide dismutase enzymes (SODs) are usually considered the first line of defence against reactive oxygen species. Two different SOD isoenzymes, contribute to superoxide anions removal inside cells. SOD1 is a copper/zinc protein located in the cytosol and in the mitochondrial intermembrane space, but also present in peroxisomes and in the nucleus. SOD2 is a mitochondrial manganese enzyme, which is the main scavenger of superoxide anions produced during the mitochondrial oxidative phosphorylation. Interestingly, both SOD1 and SOD2 transgenic mice have been demonstrated to be resistant to MPTP-induced neurotoxicity, providing evidence that some of the deleterious effects of MPTP could be mediated by superoxide radicals and strongly supporting the possibility that these radical species could play a significant role in the aetiology of PD [76, 77].

In light of these considerations, we recently evaluated the beneficial role of SODs against paraquat-induced toxicity in human neuroblastoma SH-SY5Y cell line and in *Drosophila melanogaster* [78]. Epidemiological studies demonstrated that chronic exposure to pesticides, such as PQ and rotenone, is associated with a higher risk of developing PD [79, 80]. Consistently, two independent meta-analyses found an association between pesticides, in particular PQ, and the risk of PD [81, 82]. The picture that emerges from our study emphasizes the role of both cytosolic and mitochondrial SODs in protecting cells against superoxide overproduction. Specifically, while in cells or flies treated with high concentrations of PQ (acute treatment), we observed a critical role played by SOD2 in protecting against oxidative damage, the situation observed in flies at sub-lethal concentrations of PQ (chronic treatment) indicates that only the over-expression of SOD1 is able to rescue the PQ-associated toxicity, while SOD2 appears ineffective. Interestingly, this is also true when SOD1 is specifically expressed in dopaminergic neurons [78]. Our results are in agreement with the notion that dopaminergic neurons are particularly vulnerable to oxidative conditions, and that other cytosolic processes inside these neurons, such as dopamine oxidation, may amplify the toxicity derived from an elevated production of free radical species.

Although SODs cannot be directly exploited from a therapeutic point of view, primarily for their inability to cross the BBB, still the protective effects observed with both SOD1 and SOD2 emphasize the possibility to explore the therapeutic potential of SOD mimetic compounds, i.e., small molecules possessing SOD-like catalytic properties. Four main classes of molecules possessing SOD-like activity have been described, which include metalloporphyrin, nitroxides, Mn(III)-salen complexes and Mn(II)-cyclic polyamines [83, 84]. Among them, Mn-porphyrins and Mn(II)-cyclic polyamines appear the most interesting from a chemical and therapeutic standpoint.

**Figure 2.**
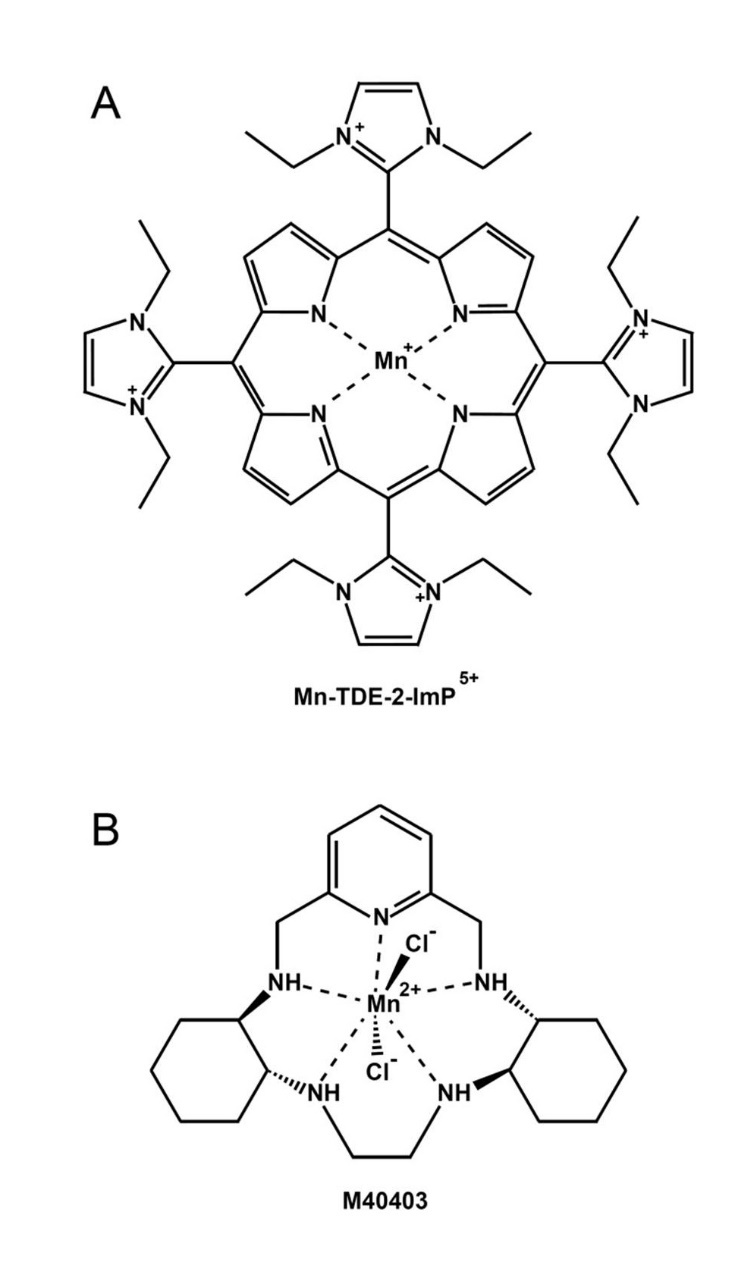
SOD-mimetic compounds (**A**) MnTDE-2-ImP^5+^, a Mn-porphyrin and (**B**) M40403, a Mn(II)-cyclic polyamine are among the most interesting SOD-mimetic molecules from a therapeutic standpoint.

### Porphyrin-based SOD mimetics

Porphyrin-based SOD mimetics include more than 50 molecules, which differ in their chemico-physical and functional characteristics. In particular, over the years many studies have sought to obtain water-soluble molecules with high catalytic activities, low toxicity [84, 85]. It is now clear that Mn-porphyrins are not simple SOD-mimetics because they also possess ONOO^-^ scavenger capability and they are able to modulate redox-sensitive cellular transcriptional activity [84]. For the reasons discussed above, the lack of specificity could represent a limit for the utilization of such molecules in clinical trials. Another possible drawback arises from a pro-oxidative activity attributed to Mn-porphyrins in the presence of cellular reductants [84]. While this feature can be therapeutically exploited in some circumstances, for example to fight cancer [86], it might make the use of Mn-porphyrins in PD more difficult. A big effort to develop new Mn-porphyrins toward clinical trial is currently being carried out and some molecules are already under clinical trials. Among them, the compound called MnTDE-2-ImP^5+^ (AEOL 10150) (Fig. 2A) resulted safe and well tolerated in a Phase I clinical trial on Amyotrophic Lateral Sclerosis patients and is now pursued as a radioprotector by the pharmaceutical company Aeolus Pharmaceuticals [87]. More importantly in the context of PD, the same company is currently working on optimization of manufacturing process and formulation of another Mn-porphyrin, AEOL 11114, which shows promise in PD, being protective in a MPTP-based mouse model of PD [88]. Very interestingly, the molecule has been demonstrated to be orally active, to have relatively long plasma half-life, rapid absorption by the gastrointestinal tract, ability to penetrate the BBB, and can achieve therapeutically active concentrations in the brain [88].

### Mn(II)-cyclic polyamines-based SOD mimetics

At the end of the last century, a new class of low molecular mass, Mn(II)-pentaazamacrocyclic ligand-based SOD mimetics has been developed to protect against inflammation in those disease-related conditions in which native SOD enzymes were found effective in animal models. This class is exemplified by the prototypical complex M40403 (Fig. 2B), a derivative of the 15-membered macrocyclic ligand 1,4,7,10,13-pentaazacyclopentadecane that contains added bis(cyclohexylpyridine) functionalities. The molecule was first tested in rat models of inflammation and ischemia-reperfusion injury showing high protection [89]. M40403 is a stable Mn(II) complex, which is resistant to oxidative degradation and is excreted intact with no detectable dissociation *in vivo*. Its catalytic rate constant has been demonstrated to be pH dependent with a rate at pH 6.0 exceeding 2×10^8^ M^-1^ s^-1^ (comparable to the native SOD2 enzymatic activity, while at pH 7.4 the rate is an order of magnitude lower (2 x 10^7^ M^-1^ s^-1^) [89]. Moreover, the molecule is water-soluble so that it can be orally administered and it is able to cross the BBB [89]. An important characteristic of M40403 is its ability to catalytically remove superoxide radicals in a very selective manner being that it does not react with other biologically relevant oxidizing species, such as, hydrogen peroxide, hypochlorite, nitric oxide and peroxynitrite [90]. Although other Mn(II)-cyclic polyamines-based SOD mimetics similar to M40403 were synthesized aimed at improving the catalytic activity while preserving its stability, until very recently only M40403 was tested in phase I and II clinical trials for the treatment of pain in approximately 700 subjects/patients, resulting in being safe and well-tolerated [91]. The pharmaceutical company Galera Therapeutic is currently pursuing the development of such a class of SOD mimetics to offer novel therapeutics to improve both the tolerability of current anti-cancer treatments and directly treat cancer. They are currently recruiting participant for a randomized Phase II clinical trial to assess the effect of their lead clinical candidate GC4419 on the incidence, severity and duration of severe oral mucositis when given to patients with squamous cell cancers of the head and neck in combination with radiation and chemotherapy (NCT02508389).

Given the positive results we obtained in our PQ-based cellular and fly models of PD and considering the aforementioned properties of M40403, we analysed its ability to prevent the PQ-induced toxicity. Not only M40403 showed significant beneficial properties in our cellular model, but, when tested *in vivo*, it succeeded in rescuing the lethality induced by elevated concentrations of PQ. Moreover, in the presence of a sub-lethal concentration of PQ, M40403 was also able to improve the locomotion behaviour of flies, a prominent aspect in the context of PD whose main clinical hallmark is represented by motor dysfunction. The protection observed with M40403, both in the presence of acute and chronic PQ treatments, is probably due to its ability to act *in vivo* both at cytosolic and mitochondrial level. Accordingly, we demonstrated that the molecule is able to rescue the toxicity induced by the down-regulation of either SOD1 or SOD2, indicating that it can act both at cytosolic and mitochondrial level [78]. This characteristic is of particular importance considering that in PD both cytosolic and mitochondrial processes could contribute to and exacerbate the production of superoxide radicals.

## Conclusions

In spite of the disappointing results obtained in the antioxidant-based clinical trials carried out until now to hamper the progression of PD, the use of antioxidant compounds could still be considered for their therapeutic potential, providing that the mechanisms underlying the oxidative injury associated to the disease are better understood. This knowledge is critical to design effective molecules able to selectively reduce the levels of defined damaging reactive species rather than eliminate ROS/RNS in a nonspecific way. A key drawback of the previously tested antioxidant therapies designed to cope with PD is that they did not specifically target what could represent the primary cause of oxidative stress, but rather the downstream effects. As several indications now exist highlighting the prominent role of the superoxide radical in inducing neuronal toxicity, a new therapeutic opportunity, to be carefully evaluated, is the use of SOD-mimetic molecules, which can selectively remove superoxide radicals in a catalytic way. The latter is a key aspect in the future prospective of these molecules: the challenge of only eliminating the ROS associated to the induce damages without interfering with the signalling activity can be address by modulating the dose going down from a proved effective concentration. Many SOD-mimetics are currently available, even though only few of them seem to have therapeutically exploitable properties. As these compounds have been initially designed to protect against inflammatory processes, a further advantage of their use is that, besides reducing oxidative damages inside cells, they might have the potential to counteract microglia chronic activation, one of the proposed cause of disease progression in PD. Nevertheless, caution must be applied when considering the protective properties of such molecules. Although porphyrin- and Mn(II)-cyclic polyamines-based SOD mimetics have shown very promising features that might be exploited to cope with PD, the experimental data describing their protective effects are mainly derived from toxin-based animal models of the disease. Unfortunately, antioxidants having potential therapeutic properties in animal models of PD very often showed disappointing results when tested in clinical trials. The main concern in this regard is that, even though a multiplicity of PD-related models exists, none of them fully recapitulate the clinical and behavioural phenotypes associated with the disease. For example, the MPTP-based mouse model has been the most used in pre-clinical studies, but the encouraging results obtained in this model were almost never reproduced in clinical trials, indicating that it is far from being a perfect model. It follows that, before initiating a new clinical study, to increase the probability of its success, different approaches should be explored, taking into consideration several experimental paradigms with the use, for example, of animal models related to both sporadic and familial forms of PD. In conclusion, with the necessary cautions, superoxide radical dismutation might represent a new promising therapeutic target in Parkinson’s disease that should be worthy of further consideration.
